# Draft Genome Sequence from a Putative New Genus and Species in the Family *Methanoregulaceae* Isolated from the Anoxic Basin of Lake Untersee in East Antarctica

**DOI:** 10.1128/MRA.00271-19

**Published:** 2019-05-02

**Authors:** Nicole Y. Wagner, Aria S. Hahn, Dale Andersen, Mary Beth Wilhelm, Connor Morgan-Lang, Mia Vanderwilt, Sarah Stewart Johnson

**Affiliations:** a Department of Biology, Georgetown University, Washington, DC, USA; b Department of Microbiology and Immunology, University of British Columbia, Vancouver, British Columbia, Canada; c Koonkie Cloud Services, Inc., Menlo Park, California, USA; d Carl Sagan Center, SETI Institute, Mountain View, California, USA; e Space Science and Astrobiology Division, NASA Ames Research Center, Moffett Field, California, USA; f Science, Technology, and International Affairs Program, Georgetown University, Washington, DC, USA; Portland State University

## Abstract

Here, we report the draft genome sequence for a new putative genus and species in the *Methanoregulaceae* family, whose members are generally slow-growing rod-shaped or coccoid methanogenic archaea. The information on this sediment-dwelling organism sheds light on the prokaryotes inhabiting isolated, deep, and extremely cold methane-rich environments.

## ANNOUNCEMENT

With methane concentrations reaching as high as 21.8 ± 1.4 mmol liter^−1^, the anoxic basin of the ice-covered Antarctic Lake Untersee is one of the most methane-rich naturally occurring aquatic ecosystems on Earth (1). An Untersee environmental sample was collected in 2016 (coordinates, 71°21′12″S, 13°26′08″E) from benthic sediments below 100 m of 4°C water using an Ekman dredge. After collection, the samples were frozen and then shipped on dry ice and stored at −80°C.

The sequence was isolated from metagenomic data. DNA was extracted using a Qiagen AllPrep kit. A paired-end library was constructed using a Kapa HyperPlus kit and sequenced on an Illumina MiSeq platform on a V3/600-cycle flow cell, generating 24,799,516 paired-end 300-bp reads for the entire metagenome. The reads were trimmed to remove adapters and bases below a quality score of 30 using Trimmomatic 0.36 (2), assembled using MEGAHIT version 1.1.2 (3), and binned using MetaBAT 0.32.5 (4). Reads were mapped back to the bin using the default parameters in Bowtie 1.2.2 (5) and SAMtools 1.3.1 (6) and reassembled using ABySS version 2.1.5 (7) to improve the metagenome-assembled genome (MAG) quality. This MAG contains 45 contigs and 1,202,444 reads (4.9% of the total metagenomic reads).

**FIG 1:**
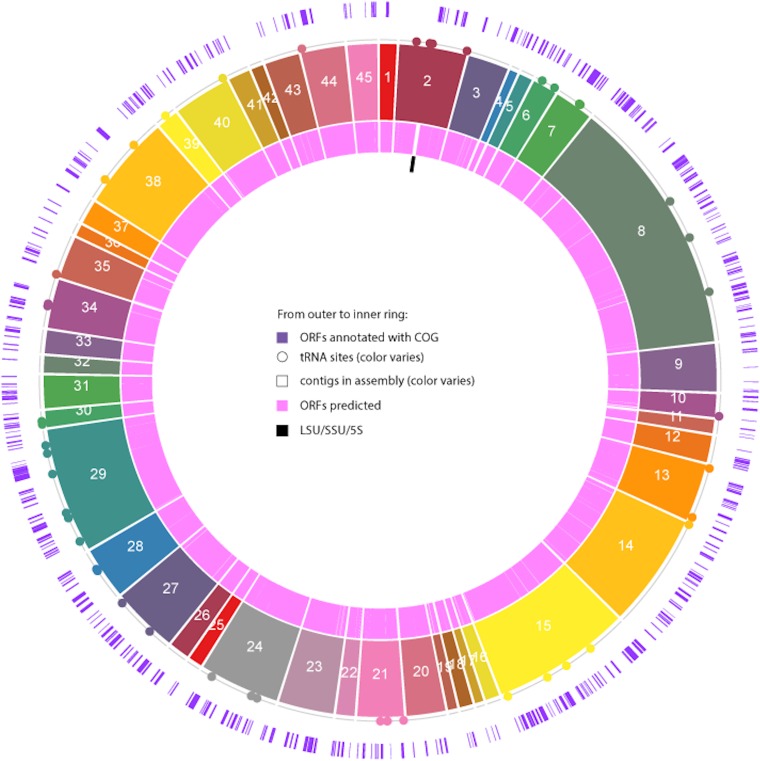
Shown from the outer to inner ring of this metagenome-assembled genome (MAG) circos plot is the following: first, the proportion of the total predicted open reading frames (ORFs) (any sequence with a start and stop codon, excluding rRNA genes) with a corresponding annotation in the COG database (17); then, tRNA locations, contigs numbered based on their order in the MAG, and all predicted ORFs. Finally, in the innermost ring, the long subunit (LSU)/SSU/5S location is shown.

The *N*_50_ value for this genome is 69,553 bp. CheckM version 1.0.11 (8) calculated completion to be 95.07% and contamination to be 0.0%. The draft genome is 2.367 Mb, with 2,318 open reading frames (ORFs), 1 small subunit (SSU) 16S rRNA gene, and 60 tRNA genes (Fig. 1). The genome was annotated using MetaPathways 2.5.1 (9) and KAAS-KEGG 2.1 (10). Default settings were used for all programs.

The 16S rRNA of this organism is 95% similar to that of Methanosphaerula palustris (11), as calculated using online BLAST-based (12) homology. This genetic distance suggests that the MAG may be classified as a previously unobserved genus in the *Methanoregulaceae* family (13).

Of the predicted ORFs in the MAG, 17.24% code for translation, ribosomal structure, and biogenesis, 10.49% belong to metabolism, and 10.53% code for amino acid metabolism genes. This MAG has a GC content of 66.4%. An average coverage of 52.261× was calculated using Bowtie 1.2.2 (5) and SAMtools 1.3.1 (6), with default parameters.

The sequence describes a methanogen with the complete *mcrABG* operon identified on a single contig. It contains the *oppA*, *mppA,* and *qseF* genes for quorum sensing and *cheA*, *cheW*, *cheR,* and *cheYB* genes associated with chemotaxis. It has ORFs for amino acid transport and synthesis, a potential response to starvation, and the regulation of nitrate uptake in low-nitrogen environments (*glnL* and *glnA* genes) (14).

The sequence also includes the *ruvABC* resolvasome genes, which are absent in Methanosphaerula palustris. These *ruvABC* genes catalyze the resolution of the Holliday junction during recombination and DNA repair (15); their presence may lead to recombination rates higher than those for Methanosphaerula palustris, as higher recombination rates have been observed to lead to higher GC content in prokaryotes (16).

### Data availability.

This whole-genome shotgun project was deposited at DDBJ/ENA/GenBank under accession number SISS00000000, and the reads were deposited under accession number PRJNA521775. This study is version SISS01000000.
